# Interpreting Non-Inferiority Across Effect Scales: A Bayesian Perspective with Clinical Translation

**DOI:** 10.1007/s43441-026-00982-4

**Published:** 2026-05-14

**Authors:** Danila Azzolina, Silvia Bressan, Mohd Rashid Khan, Dario Gregori, Savino Spadaro

**Affiliations:** 1 Biostatistics and Clinical Trial Biometry, Clinical Research Center DEMeTra, Department of Translational Medicine, University of Naples Federico II, Naples, Italy; 2 Paediatric Emergency Division, Department of Women and Children’s Health, University of Padova, Via Giustiniani 3, 35128 Padova, Italy; 3 Unit of Biostatistics, Epidemiology, and Public Health, Department of Cardiac, Thoracic, Vascular Sciences, and Public Health, University of Padua, Padua, Italy; 4 Department of Translational Medicine, Unversità Degli Studi Di Ferrara, Ferrara, Italy

**Keywords:** Non-inferiority trials, Bayesian trials, Paediatrics, Critical care, Absolute and relative risk measures, Number needed to harm (NNH), Clinical interpretation of margins

## Abstract

**Objective:**

This study examines how the choice of non-inferiority margins affects the clinical interpretation of interventional studies using the Santos et al. trial comparing high-flow nasal cannula (HFNC) with non-invasive ventilation (NIV) as a case example. We also demonstrate how Bayesian reanalysis improves interpretability by expressing non-inferiority margins on absolute and relative scales, quantifying posterior probabilities of non-inferiority and benefit, and translating these margins into patient-centred measures such as the Number Needed to Harm (NNH).

**Design:**

Secondary Bayesian reanalysis of a previously published randomised controlled trial.

**Setting:**

Multicentre paediatric intensive care units participating in the original Santos et al. study.

**Patients:**

Infants with acute bronchiolitis were randomised to receive either HFNC or NIV.

**Interventions:**

Respiratory support via HFNC or NIV.

**Main Outcome Measures:**

The primary endpoint was intubation, with the intubation rate reported as a descriptive summary statistic. We re-expressed the original non-inferiority margin on the Absolute Risk Reduction (ARR) and Odds Ratio (OR) scales, estimated Bayesian probabilities of non-inferiority and benefit, and derived patient-centred metrics such as the NNH.

**Results:**

The original analysis concluded non-inferiority using a fixed absolute margin. Our Bayesian reanalysis showed that this margin could correspond to a substantial relative tolerance when baseline risks are low; specifically, the 0.15 non-inferiority ARR margin might imply a doubling of the odds of intubation. When interpreted from a patient-centered perspective, this margin corresponds to an NNH of approximately 6.7. In other words, for every seven infants treated with HFNC instead of NIV, one additional child might require intubation who would not have needed it under standard care.

**Conclusions:**

Non-inferiority margins are typically derived through a combination of statistical evidence and clinical judgment. However, their interpretation may vary depending on the effect scale used, and this may not always be transparent in reporting. For this reason, integrating Bayesian methods and framing multiple noninferiority definitions improves the interpretability of trials.

**Supplementary Information:**

The online version contains supplementary material available at https://doi.org/10.1007/s43441-026-00982-4.

## Introduction

Non-inferiority trials are often conducted to evaluate interventions that are easier to administer, less invasive, or less resource-intensive than standard care [1].

Non-inferiority in clinical trials means showing that a new treatment is not unacceptably worse than an established standard by more than a pre-specified margin (Δ) [2]. This margin reflects the most significant difference in the primary outcome, often an increase in adverse events or reduction in efficacy, that clinicians and patients would consider clinically tolerable, given potential advantages of the new therapy (e.g., safety, cost, ease of use) [3].

In the literature, the non-inferiority margin is generally expressed on an absolute scale, for example, as Absolute Risk Reduction (ARR) or Mean Difference (MD). Defining non-inferiority solely in terms of an absolute difference can mask substantial relative increases in harm, particularly in populations with a low baseline risk profile [4]. When baseline risk is low, a fixed absolute non-inferiority margin can imply a large and clinically unacceptable relative increase in harm. For example, with a 10% control event rate, allowing a 15% absolute increase permits a rise to 25%, corresponding to a 150% relative increase in risk, which may be statistically acceptable yet clinically alarming. Such a conclusion, though statistically permissible, may be ethically concerning because non-inferiority margins that allow a clinically meaningful loss of benefit risk declare an intervention non-inferior while accepting outcomes that may be worse for patients.

In non-inferiority trials, margins are defined using established frameworks that integrate statistical evidence and clinical judgment. However, even when appropriately specified, the interpretation of these margins may depend on the effect measure used (e.g., absolute or relative scale), which can lead to differences in how the results are understood [5]. Similarly, Cuzick [3] highlighted the risks of relying solely on absolute margins. In the TARGIT-A trial, the 2.5% absolute non-inferiority margin was met despite a relative risk of recurrence exceeding 2.0, raising concerns about clinical acceptability. Furthermore, Althunian et al. [6] argue that non-inferiority margins should be chosen and interpreted with careful consideration of absolute and relative scales.

These concerns indicate that absolute-only non-inferiority margins can make it difficult to interpret clinically meaningful harm. This issue is, for example, evident in pediatric critical care, where even small increases in adverse events, such as intubation, have significant implications [7].

The Santos et al. trial [8] provides a real-world case study highlighting the clinical and methodological challenges in defining non-inferiority margins. The primary outcome was the intubation among children receiving either high-flow nasal cannula (HFNC) or non-invasive ventilation (NIV) for acute hypoxemic respiratory failure due to bronchiolitis [8]. HFNC may offer comparable or slightly reduced effectiveness relative to NIV in preventing intubation. Still, it confers practical advantages, including greater infant tolerance, ease of administration, and lower resource demands [9]. The trial, for example, used a non-inferiority margin of 15% in ARR [8], allowing HFNC to be considered non-inferior if the increase in intubation rate did not exceed this threshold relative to NIV. In this research framework, the baseline risk of intubation is relatively low (7–10%) according to the literature [5]; for this reason, a fixed absolute noninferiority margin could result in a disproportionately large relative increase in harm [5].

Even if this work is motivated by a pediatric critical care trial, the methodological issues discussed here are not specific to pediatrics only. Fixed absolute non-inferiority margins can lead to clinically misleading interpretations in any setting where baseline event rates are low or vary from those anticipated at the design stage [5]. Pediatric critical care is considered in this manuscript as a clinically meaningful example because even modest increases in invasive outcomes, such as intubation, may carry disproportionate clinical and ethical implications [7].

Within this general framework, Bayesian methods also offer a promising approach. A Bayesian trial is a clinical study that uses Bayesian statistical methods to continuously update the probability of treatment benefit or harm as data accumulate [10]. Non-inferiority inference is inherently threshold-based, requiring assessment of whether treatment effects fall within a prespecified margin. In this context, Bayesian approaches enable direct estimation of the posterior probability that the treatment effect satisfies the non-inferiority criterion, aligning naturally with the underlying clinical question. Moreover, they allow coherent evaluation across different effect scales and support probabilistic statements about clinically meaningful quantities, such as the probability of exceeding margins defined; Bayesian trials provide direct, intuitive probability statements, such as the probability a treatment is non-inferior [11], e.g. “there is a 99% probability that the treatment meets the non-inferiority threshold”.

Bayesian approaches facilitate also the interpretation of treatment effects, enabling results to be reported in absolute and relative terms. Posterior distributions can be used to derive ARR in absolute terms, OR in relative scale, and NNH within a unified framework, fostering more transparent, patient-centered communication [11]. In particular, margins defined on ratio scales may not directly convey the potential impact on patients. To address this gap, the NNH is a complementary measure that translates non-inferiority margins and treatment effects into an absolute, patient-centered metric. By expressing the maximum acceptable loss of efficacy as the number of patients who would need to be treated for one additional adverse outcome to occur, NNH provides a direct link between statistical inference and clinical decision-making.

Moreover, while the importance of interpreting non-inferiority on absolute and relative scales is recognized, current practice lacks a coherent inferential framework that links scale choice, non-inferiority margins, and clinical interpretation; for this reason, we propose a unified framework that formalizes how conclusions may differ across scales and demonstrates how Bayesian inference provides a natural mechanism for quantifying and reconciling these discrepancies. This approach aims to bridge the gap between methodological recommendations and practical decision-making. This research presents a Bayesian reanalysis of the Santos et al. trial [8] as a motivating example, underscoring the implications of interpreting the non-inferiority margin on multiple scales.

Methods

## Methods

### Absolute Versus Relative Margins: Clinical Meaning Matters

Non-inferiority margin on the relative scale (OR) was derived by transforming the absolute margin under a fixed reference (control) event rate. Specifically, the maximum acceptable event rate in the experimental group was defined as the control event rate plus the absolute non-inferiority margin, and the corresponding OR was computed from these quantities. Specifically, the probability of the adverse event in the control arm is $\:{\mathrm{p}}_{\mathrm{c}}$ and the corresponding odds are $\text { odds }_c=\frac{p_c}{1-p_c}$. If we allow an absolute non-inferiority margin of $\:{\Delta\:}$, the event probability in the treatment arm is assumed to be at most $\:{p}_{t}={p}_{c}+{\Delta\:}$, and the corresponding odds are $\:{\mathrm{odds}}_{t}=\frac{{p}_{c}+{\Delta\:}}{1-\left({p}_{c}+{\Delta\:}\right)}$. The OR margin is defined as the ratio of treatment to control odds:

$$\:{{\Delta\:}}_{OR}=\frac{{\mathrm{odds}}_{t}}{{\mathrm{odds}}_{c}}=\frac{\frac{{p}_{c}+{\Delta\:}}{1-\left({p}_{c}+{\Delta\:}\right)}}{\frac{{p}_{c}}{1-{p}_{c}}}$$

This expression shows that the same absolute margin increase ($\:{\Delta\:}$) translates into different relative odds depending on the baseline event rate ($\:{\mathrm{p}}_{\mathrm{c}}$​).

- If $\:{\mathrm{p}}_{\mathrm{c}}$​ is high, the relative increase appears smaller.
- If $\:{\mathrm{p}}_{\mathrm{c}}\:$is low, the same absolute increase produces a much larger OR, magnifying the perceived harm.

To improve clinical interpretation, the absolute non-inferiority margin can also be translated into the NNH, representing how many patients would need to be treated with the new intervention for one additional adverse outcome to occur if the margin were fully realized. It is calculated as$\:\:\mathrm{N}\mathrm{N}\mathrm{H}=\frac{1}{{\Delta\:}}$ where $\:{\Delta\:}$ is the absolute ARR non-inferiority margin expressed as a proportion.

**Fig. 1:**
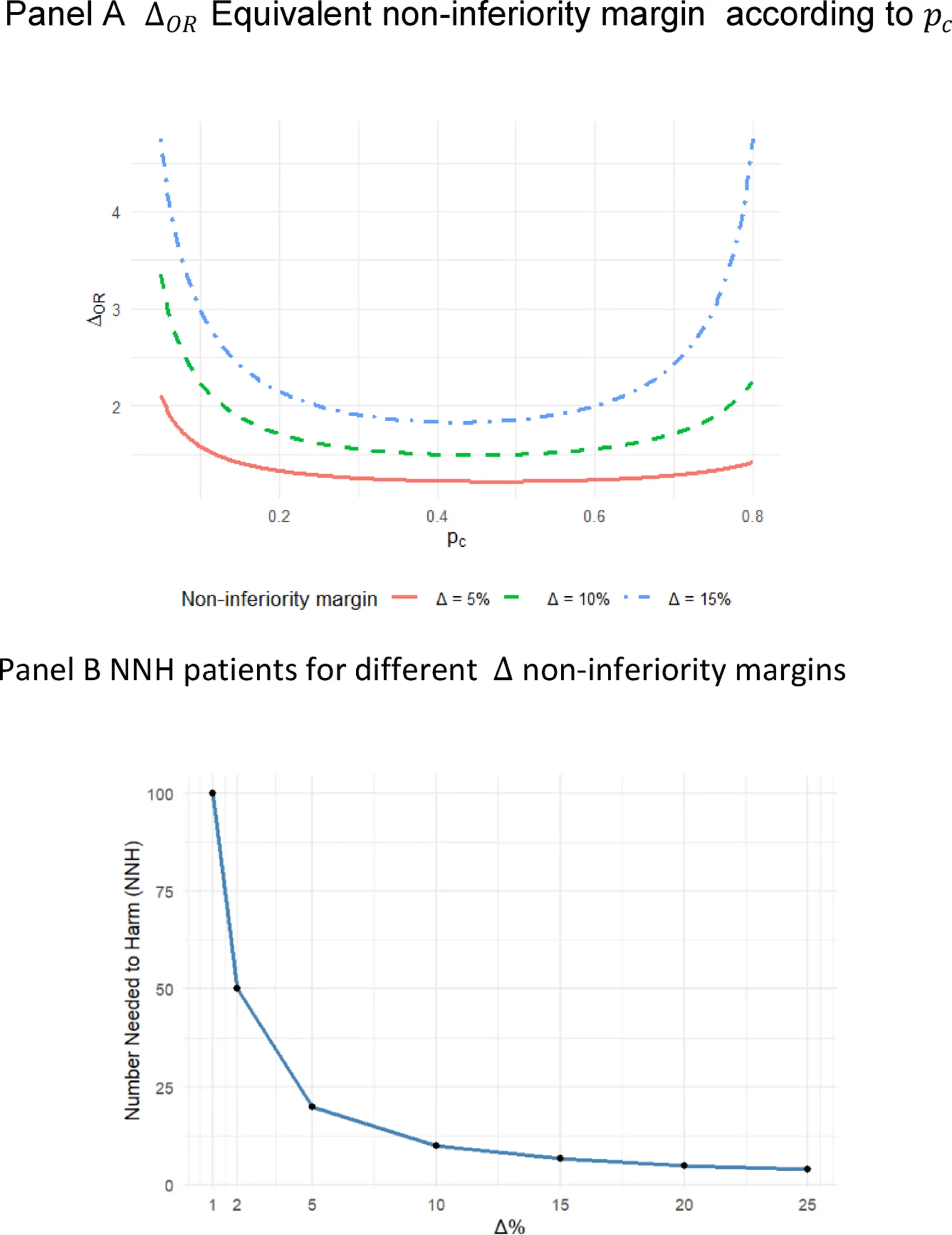
OR Equivalent non-inferiority margin $\:{{\Delta\:}}_{OR}$ according to different control or standard of care event rate $\:{p}_{c}\:$ (Panel A), and NNH patients for different $\:{\Delta\:}$ non-inferiority margins (Panel B)

The relation with the absolute value non-inferiority margin Δ and the $\:{{\Delta\:}}_{OR}$ and NNH is reported in Fig. 1.

Fixed absolute risk differences, which are often used to define non-inferiority margins, can appear deceptively acceptable. However, their impact depends strongly on the baseline event rate. As the baseline risk decreases, the same absolute margin translates into much larger relative risks (Fig. 1 Panel A). For instance, a 0.15 ARR margin corresponds to an OR greater than 3 when the control event rate is only 10%. In practical terms, this means the new intervention could more than triple the odds of a harmful outcome, such as intubation, and still be deemed “non-inferior” under such a margin.

Panel B illustrates this point from a patient-impact perspective using the Number Needed to Harm (NNH). With a 1% absolute margin, the NNH is 100, meaning one extra child would be harmed for every 100 treated, a relatively small risk. By contrast, with a 0.15 ARR non-inferiority margin, the NNH falls dramatically to just 6–7, meaning one additional child would be harmed for every 6–7 treated. Clinically, this represents an unacceptably high proportion of patients.

**Fig. 2:**
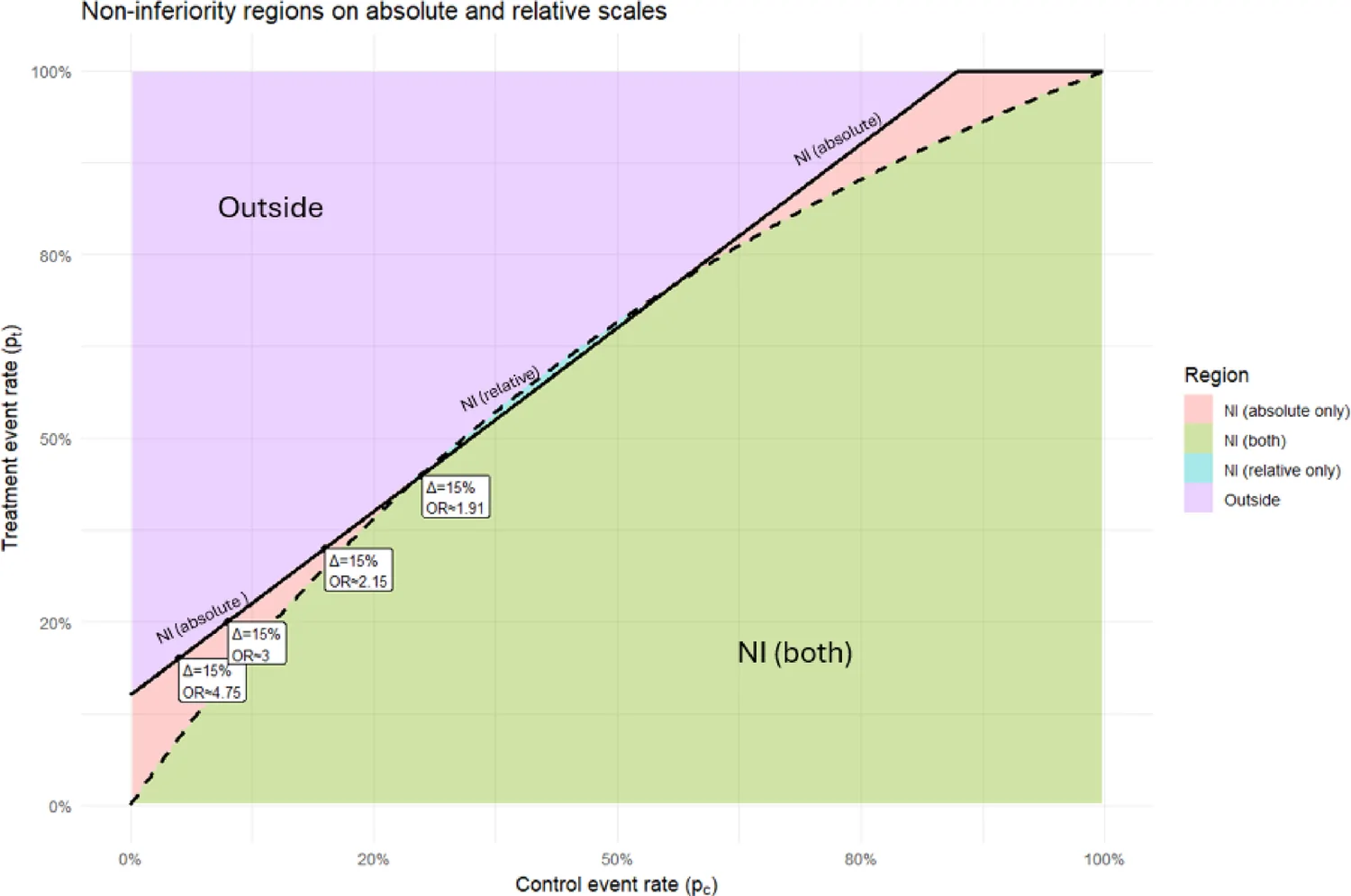
Two-dimensional representation of non-inferiority regions defined on absolute and relative scales. The x-axis shows the control event rate ($\:{p}_{c}$) and the y-axis the treatment event rate ($\:{p}_{t}$). The solid line represents the absolute non-inferiority margin ($\:{p}_{t}\le\:{p}_{c}+{\Delta\:}$, with $\:{\Delta\:}=0.15$). In contrast, the dashed line represents the corresponding odds-ratio margin. Shaded regions indicate scenarios meeting non-inferiority on the absolute scale only, the relative scale only, both scales, or neither. Annotated points illustrate how a fixed absolute margin corresponds to increasingly large relative effects as baseline risk decreases

Figure 2 illustrates the non-inferiority regions defined on absolute and relative scales across combinations of control ($\:{p}_{c}$) and treatment ($\:{p}_{t}$) event rates. When baseline risk is low, the absolute margin ($\:{\Delta\:}=0.15$) allows treatment event rates corresponding to large OR, whereas the absolute and relative non-inferiority boundaries progressively converge as the control event rate increases. The shaded regions indicate scenarios in which non-inferiority holds only on the absolute scale, only on the relative scale, on both scales, or on neither scale.

We formalize a general approach to interpret non-inferiority margins by mapping an absolute margin (Δ) to (i) its corresponding OR as a function of baseline risk and (ii) the NNH. Because the relationship between absolute and relative measures depends on baseline risk and is nonlinear, a single margin defined on one scale does not correspond to a unique inferential threshold on another scale. This framework is independent of the specific case study and can be applied to any non-inferiority setting.

### Reframing the Margin: A Bayesian Reanalysis of the Case Study

This manuscript uses the Santos et al. trial as an illustrative example to demonstrate how fixed absolute non-inferiority margins can translate into large relative and patient-level harms when baseline risks are low [8]. This example is presented for illustrative purposes to demonstrate scale-dependent interpretation and is not intended to endorse the clinical appropriateness of the selected non-inferiority margin.

The trial’s primary aim was to assess whether HFNC therapy is non-inferior to noninvasive ventilation (NIV/BiPAP) in children with bronchiolitis and respiratory failure, using the intubation as the primary outcome [8]. The authors hypothesized that HFNC would not result in a higher intubation rate beyond a predefined non-inferiority margin of 0.15 ARR compared to NIV.

We do not suggest that a 0.15 ARR margin for intubation is clinically unacceptable; rather, this example illustrates how margins that meet formal non-inferiority criteria may nonetheless permit levels of harm that most clinicians would find unacceptable when translated into absolute, relative, and patient-centred terms.

We reanalysed the data using the publicly available data on Dryad (https://doi.org/10.5061/dryad.4xgxd25jh), with a Bayesian approach to evaluate the primary outcome of intubation.

This study represents a secondary reanalysis of a previously published randomised controlled trial. While no specific EQUATOR guideline exists for reanalyses, we adhered to the general principles of transparency in reporting trial analyses. We followed the SAMPL guidelines to ensure precise and reproducible presentation of statistical methods and results [12] (Table S1).

### Analysis

We specified a Binomial distribution with a logit link for modelling intubation risk in a Bayesian Logistic regression model. We used weakly informative Normal(0, 2.5) priors on the log-odds scale for the intercept and regression coefficients. This choice provides mild regularization while allowing a broad range of clinically plausible effects (corresponding to OR approximately between 0.08 and 12 within the central 95% prior mass). The Bayesian approach quantifies uncertainty and allows probability-based interpretation expressed on the computed posterior distribution as:

1. The probability that HFNC is non-inferior to NIV P(ARR < Δ).
2. The probability that HFNC is non-inferior on the OR scale (OR < Δ_OR_).
3. The probability that HFNC is superior to NIV in reducing intubation, $\:\mathrm{P(ARR<}\mathrm{0}\mathrm{)}$ and $\:\mathrm{P(OR<}\mathrm{1}\mathrm{)}$.

We express relative effects on the OR scale because the Bayesian logistic regression model naturally estimates treatment effects in terms of log-odds, allowing a direct and internally consistent transformation of the absolute non-inferiority margin.

Posterior distributions of treatment effects were summarized OR for HFNC versus NIV, with 95% highest density intervals (HDIs). To account for non-inferiority margins defined on absolute and relative scales, we derived the transformation linking the absolute risk difference margin (Δ) to its OR Δ_OR_ equivalent as a function of the baseline risk in the NIV group $\:{p}_{c}$. We visualized the posterior distributions of ARR and OR, shading the non-inferiority zones to illustrate the proportion of posterior mass supporting the non-inferiority hypothesis. This definition does not imply equivalence in posterior probabilities across scales, as such equivalence is not generally preserved under nonlinear transformations.

To improve clinical interpretability, we also expressed the absolute margins as NNH.

Four Markov chains were run in parallel across 2000 iterations to ensure that the sampling algorithm converged to the same posterior distribution regardless of the starting point; agreement across chains indicates stable, reliable posterior estimates. Following standard practice to assess convergence and between-chain agreement.

Convergence was evaluated using standard diagnostics, including trace plots.

Analyses were performed with R 3.4.2 [13] and rstanarm [14] package.

## Results

In the original trial analysis, 37/89 patients in the NIV group (29%) and 29/97 patients in the HFNC group (23%) required intubation (*p* = 0.25). Using the Farrington-Manning test for non-inferiority with a predefined 0.15 ARR non-inferiority margin, the absolute difference in intubation rates was 6.3% in favor of HFNC (95% CI: − 4.5 to 17.1%), meeting the statistical threshold for non-inferiority (*p* < 0.0001)) [8].

**Fig. 3:**
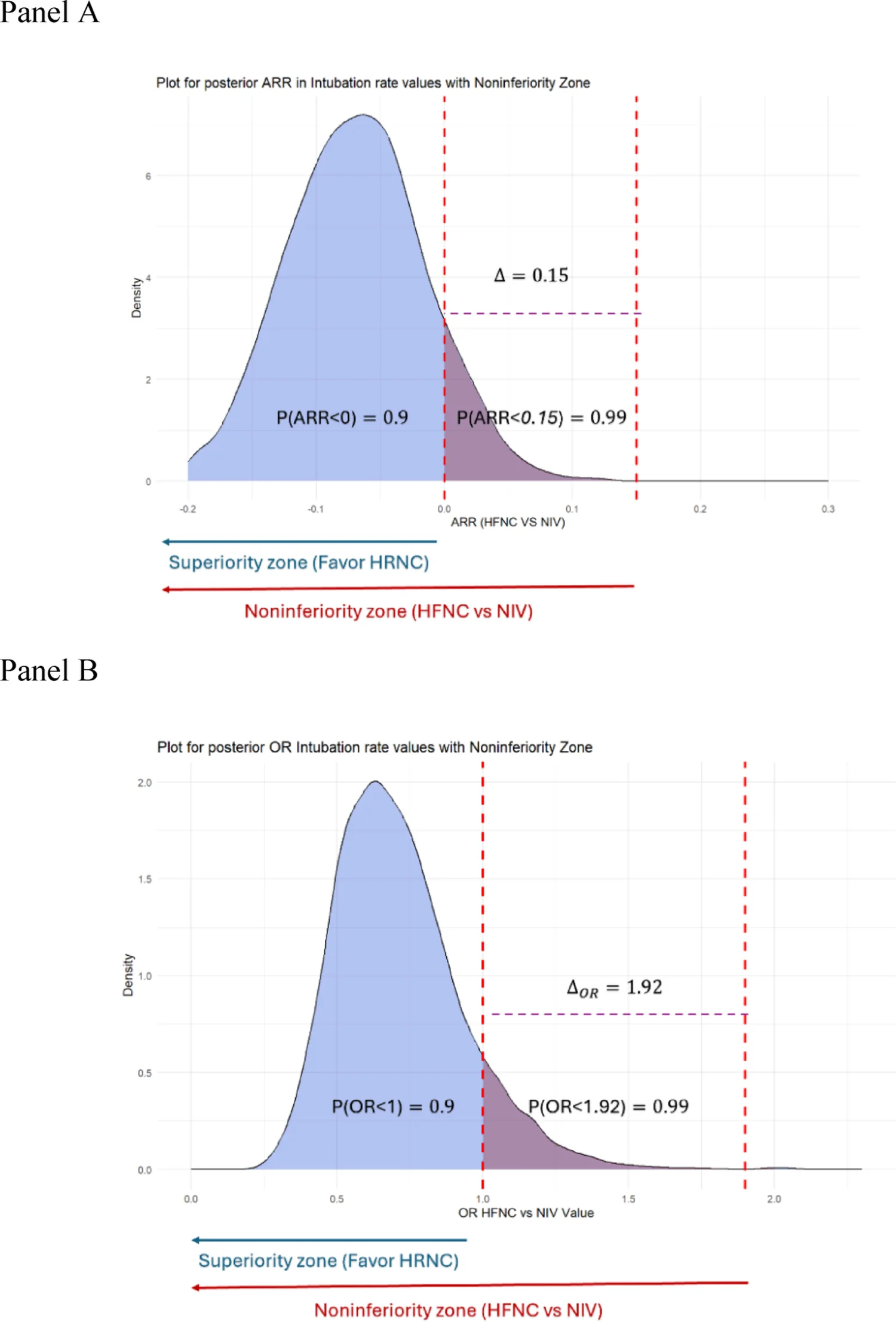
Bayesian posterior distributions of the treatment effect comparing high-flow nasal cannula (HFNC) to non-invasive ventilation (NIV) for intubation risk in children with bronchiolitis. Panel A displays the posterior distribution of the absolute risk reduction (ARR) in intubation (HFNC vs. NIV). The dashed red lines mark the non-inferiority margin (Δ = 0.15). The shaded blue area shows the probability that HFNC is superior (ARR < 0), and the combined shaded blue and purple area shows the probability that HFNC is non-inferior (ARR < 0.15). Panel B shows the posterior distribution of the odds ratio (OR) for intubation (HFNC vs. NIV). The red dashed line at OR = 1 denotes no effect, while the non-inferiority margin corresponds to OR = 1.92. The shaded blue area represents the probability that HFNC reduces intubation risk (OR < 1), and the combined shaded region represents the probability of non-inferiority (OR < 1.92)

In our reanalysis, the posterior distribution for the absolute risk difference (ARR) in intubation rate between HFNC and NIV is reported in Fig. 3. The shaded blue area to the left of zero reflects the probability that HFNC is superior to NIV, which is estimated at 90%. The shaded purple area under the curve to the left of the 15% non-inferiority threshold represents the probability that HFNC is non-inferior, calculated as 99% (Fig. 3, Panel A). This framing not only confirms non-inferiority but also demonstrates a high likelihood of benefit, thereby reinforcing confidence in the clinical interpretation beyond the binary conclusion of a traditional p-value.

Panel B shows the same analysis expressed on the relative scale, using OR. Here, the posterior distribution of the OR for intubation with HFNC versus NIV again shows a 90% probability that the actual OR is below 1, indicating a likely benefit of HFNC. The purple-shaded area to the left of the OR-based non-inferiority threshold of 1.92 indicates a 99% probability that the odds of intubation with HFNC do not exceed this margin, thereby satisfying the criterion for non-inferiority. The non-inferiority margin was defined as a 15% absolute increase in the risk of intubation with HFNC compared to NIV. With an observed intubation rate of 29% in the NIV group, this means HFNC would be considered non-inferior even if its intubation rate reached 44% (i.e., 29% + 15%).

When translated into relative terms, the OR non-inferiority threshold is obtained by transforming the absolute margin under the observed control event rate by approximately 1.92. That is, HFNC would be permitted to nearly double the odds of intubation compared to NIV and still be declared non-inferior. This transformation reflects a change of scale rather than an equivalence of inferential conclusions, and differences in posterior probabilities across scales are expected.

When interpreted from a patient-centered perspective, this margin corresponds to an NNH of approximately 6.7. In other words, for every seven infants treated with HFNC instead of NIV, one additional child might require intubation who would not have needed it under standard care.

All chains converged to the same posterior distribution, as confirmed by visual inspection of trace plots and standard diagnostics (Fig. S1).

## Discussion

Our Bayesian reanalysis of the Santos et al. trial [8], a non-inferiority RCT comparing HFNC with NIV in infants with bronchiolitis, corroborates the finding that HFNC is not worse than NIV on the prespecified margin. The magnitude of the non-inferiority margin in the pediatric example, as well as its clinical acceptability, should be evaluated within established regulatory frameworks [6]. In this work, the example is used solely to illustrate how interpretation may vary across effect scales.

Non-inferiority margins should be formally specified on a single, protocol-defined scale. However, the implications of this margin should be evaluated and reported on alternative scales through appropriate transformations based on the baseline risk. Relative measures tend to be more stable across varying baseline risks, whereas absolute measures are often more directly relevant for clinical decision-making and patient-level interpretation. In this context, margins on secondary scales are derived quantities rather than independently specified thresholds. These perspectives are complementary rather than competing, and their joint reporting may improve transparency and support more informed clinical decisions. In particular, absolute measures such as Number Needed to Harm provide a direct translation of results into patient-relevant consequences.

In pediatric critical care, increasing invasive procedures, such as intubation, can have lasting consequences [15]. When a non-inferiority margin allows an intervention to increase the risk of intubation by up to 15% points, this may correspond to a nearly doubling of the odds of harm and an NNH as low as 6 to 7 [15]. Unlike conventional effect measures, NNH provides an intuitive, decision-oriented metric that is readily understood by clinicians and stakeholders. Moreover, because NNH depends on baseline risk, it naturally reflects the same scale-dependence that underlies differences between absolute and relative measures, further reinforcing the need for multi-scale interpretation of non-inferiority results.

Although non-inferiority margins are formally defined using established methodological principles informed by clinical judgment and historical evidence, their clinical implications may be obscured when fixed absolute margins are interpreted without explicit consideration of baseline risk or corresponding relative and patient-centred effects [6]. Non-inferiority claims, when misinterpreted as evidence of equivalence or safety, may lead to the premature adoption of strategies that are easier to use but significantly less effective [5]. The potential for misinterpretation is relevant, for example where even small increases in risk can carry disproportionate clinical and ethical consequences [3], and where the threshold for what can be considered tolerable harm must therefore remain especially stringent [16]. However, while pediatric critical care provides a compelling example, the considerations raised here extend to non-inferiority trials in other clinical fields, particularly when outcomes are rare but clinically severe.

Moreover, the relationship between absolute and relative effect measures is bidirectional and depends on the baseline event rate. While small absolute risk differences may correspond to large relative risks or odds when event rates are low, the converse is also true: large relative effects can translate into small absolute risks in low-risk settings. This latter perspective has been widely used, particularly in cardiovascular research, to motivate the use of absolute risk measures or to justify relatively small non-inferiority margins despite seemingly large relative effects. Accordingly, the choice of scale for defining non-inferiority margins should be guided by clinical relevance, baseline risk, and interpretability, rather than by the magnitude of the effect measure alone [17].

From a reporting and regulatory standpoint, our approach is consistent with contemporary guidance. The CONSORT [18] extension for non-inferiority/equivalence trials emphasizes clear depiction of the margin, the analysis scale, and interval estimates, and the FDA’s updated guidance reiterates careful margin justification, sensitivity to historical evidence, and transparent communication of clinical acceptability [19].

Moreover, when non-inferiority margins are communicated only as absolute percentages, their implications remain opaque to both clinicians and families. Translating margins into interpretable metrics, such as the number of additional children harmed per 100 treated (NNH) or the relative odds of harm, enables more transparent and ethically sound communication. Saying “this margin allows a 90% increase in intubation risk” or “one in seven children may be harmed” is more aligned with real-world decision-making than “the difference was within 15% absolute increase.”

While the advantages of Bayesian inference are general, they are particularly well aligned with non-inferiority trials, where the primary objective is to quantify the probability that a treatment effect lies within an acceptable margin. This enables direct, decision-oriented interpretation of results that is less naturally obtained within a frequentist framework. Non-inferiority inference is inherently threshold-based, requiring evaluation of whether treatment effects exceed a prespecified margin [10]. In this setting, Bayesian approaches allow direct quantification of the posterior probability that the treatment effect lies within the non-inferiority region, which aligns naturally with the clinical question of interest. In addition, Bayesian methods facilitate the evaluation of multiple thresholds across different effect scales and enable direct probabilistic statements about clinically relevant quantities, such as the probability of exceeding a margin expressed in absolute or relative terms [20].

Non-inferiority margins and decision thresholds should be formally specified on a single scale to preserve statistical coherence. However, the implications of these choices should be evaluated and interpreted across alternative scales to ensure clinical relevance and avoid misleading conclusions. By quantifying the posterior probability that an intervention exceeds prespecified thresholds, Bayesian methods allow clinicians to directly assess the likelihood that a new treatment causes clinically relevant harm. This probability-based reporting aligns more closely with clinical reasoning than traditional p-value-based approaches, as clinicians naturally think in terms of risks and likelihoods [10, 20]. Moreover, Bayesian interpretation, reporting results as absolute risk differences/NNH, as OR or relative risks, responds to calls in the methodological literature for margins to be justified [5, 21]. This approach also supports adaptive trial monitoring, allowing for early stopping due to futility or harm when interim data indicate a high likelihood that the margin will be exceeded [21]. Bayesian hierarchical models also enable information sharing across sites, improving efficiency in multicenter trials, particularly important in pediatrics, where recruitment is often slow and sample sizes are small.

Our aim is not to introduce a new critique, but to operationalize existing methodological principles into a form that is directly usable in trial interpretation and reporting. Moreover, we do not advocate for a formal requirement that non-inferiority be demonstrated simultaneously on multiple scales. We note that formally requiring non-inferiority to be shown on multiple scales would pose substantial challenges for frequentist power calculations and hypothesis testing, including issues of multiplicity and type I error control. For this reason, we do not advocate multiple formal non-inferiority tests, but rather a single prespecified primary analysis scale, complemented by translation of the chosen margin in a relative scale to support transparent clinical interpretation and ethical judgment. This distinction is relevant when translating results into clinical decision-making, where different scales may convey different impressions of the magnitude of acceptable loss of efficacy.

## Take-Home Message

- Our findings do not question the clinical grounding of non-inferiority margins, which are typically defined through collaboration between statisticians and clinicians and supported by regulatory guidance. Rather, our results highlight that, even when margins are appropriately specified, their interpretation can vary across effect scales. In critically ill children, even small absolute increases in adverse outcomes may represent a burden that families and clinicians would not tolerate.
- Non-inferiority margins should be specified on a single, protocol-defined scale. However, results should be presented on both absolute and relative scales to reflect both statistical stability and clinical interpretability. In this context, margins on secondary scales are not independently specified but are derived from the primary margin.
- Third, results should be translated into patient-centered metrics such as the NNH. Saying that “one in every seven children might experience an avoidable intubation” conveys the clinical stakes far more clearly than reporting a 15% risk difference. These translations also make discussions with families and caregivers more transparent and ethically grounded.
- Fourth, Bayesian methods offer advantages by allowing probability statements such as “there is a 90% chance the intervention is noninferior” which align more closely with the way clinicians and families interpret risk.

## Conclusions

This reanalysis highlights that the definition and communication of non-inferiority margins play a decisive role in shaping their clinical interpretation. Reliance on a fixed absolute margin can mask clinically significant relative risks, an issue of particular concern in pediatrics, where the threshold for acceptable harm must remain especially stringent.

By reframing results, accounting for relative scales, and translating them into patient-centered terms such as the NNH, clinicians and families gain a clearer understanding of what “non-inferior” means in practice. The Bayesian framework adds further value by providing probability-based statements that align with clinical reasoning and allow more transparent communication of uncertainty. Our framework is intended to complement, rather than replace, existing approaches for defining non-inferiority margins and regulatory guidance.

## Supplementary Information

Below is the link to the electronic supplementary material.


Supplementary Material 1



Supplementary Material 2


## Data Availability

We reanalysed the data using the publicly available data on Dryad (https://doi.org/10.5061/dryad.4xgxd25jh).
