# Supplementary material for: Interpreting Non-Inferiority Across Effect Scales: A Bayesian Perspective with Clinical Translation
Source: Ther Innov Regul Sci. 2026 May 14;60(5):1419–28. doi: 10.1007/s43441-026-00982-4 (PMC13526082; doi:10.1007/s43441-026-00982-4)
Supplement: Supplementary file 1 [file 43441_2026_982_MOESM1_ESM.docx]

**Table S1. SAMPL-adapted checklist for transparent statistical reporting in this reanalysis.**
This checklist, adapted from the Statistical Analyses and Methods in the Published Literature (SAMPL) guidelines, documents how reporting standards were applied throughout the manuscript to ensure clarity, reproducibility, and clinical interpretability.

| Section | Item | Recommendation | Reported in manuscript section |
| --- | --- | --- | --- |
| Study Design & Objectives | 1 | State that this is a **secondary Bayesian reanalysis** of a published RCT | Introduction, Methods (Design) |
|  | 2 | Clearly define the **objective** (re-examining non-inferiority margins using Bayesian framework, comparing absolute vs relative scales) | Introduction, Methods (Objectives) |
| Data Description | 3 | Describe the **original dataset** (trial name, patient population, outcomes) | Methods (Data source) |
|  | 4 | Specify any **synthetic dataset** used for sensitivity checks | Methods (Statistical analysis) |
|  | 5 | Define all **variables and outcomes** (e.g., intubation, trial duration, covariates) | Methods (Variables) |
| Statistical Methods | 6 | Identify the **models used** (Bayesian logistic regression, Bayesian model for trial duration) | Methods (Statistical analysis) |
|  | 7 | State the **link function** (logit) | Methods (Statistical analysis) |
|  | 8 | Report the **priors** (Normal(0, 2.5) for coefficients and intercepts) | Methods (Statistical analysis) |
|  | 9 | Describe how **posterior distributions** were summarized (means, HDIs) | Methods (Statistical analysis) |
|  | 10 | Explain **non-inferiority margins** on absolute and relative scales, with formula | Methods (Statistical analysis) |
|  | 11 | Describe use of **Number Needed to Harm (NNH)** for clinical interpretation | Methods (Statistical analysis) |
| Statistical Presentation | 12 | Present **numerical effect sizes** (ORs, ARRs) with **95% credible intervals** | Results (Main findings) |
|  | 13 | Report **posterior probabilities** of non-inferiority | Results (Posterior analysis) |
|  | 14 | Provide **visualizations** (posterior density plots, non-inferiority regions) | Results (Figures 1–2) |
|  | 15 | Clarify **probability statements** (e.g., “X% probability of non-inferiority”) | Results, Discussion |
| Interpretation & Transparency | 16 | Discuss potential **confounding and limitations** of the reanalysis | Discussion (Limitations) |
|  | 17 | Explain **clinical meaning** of margins (absolute, relative, NNH) | Discussion (Clinical interpretation) |
|  | 18 | Distinguish **statistical non-inferiority** from **clinical acceptability** | Discussion |
|  | 19 | State explicitly that the reanalysis does not challenge the **validity of the original trial results** | Discussion (Contextualization) |
| Reporting Standards | 20 | Declare adherence to **CONSORT principles** for transparency | Methods (Reporting) |
|  | 21 | Declare adherence to **SAMPL guidelines** for statistical reporting | Methods (Reporting) |
|  | 22 | Provide reproducibility details (software: R, rstanarm, bayestestR, marginaleffects, seed set) | Methods (Software, reproducibility) |
