# Supplementary material for: Interpreting Non-Inferiority Across Effect Scales: A Bayesian Perspective with Clinical Translation
Source: Ther Innov Regul Sci. 2026 May 14;60(5):1419–28. doi: 10.1007/s43441-026-00982-4 (PMC13526082; doi:10.1007/s43441-026-00982-4)
Supplement: Supplementary file 2 [file 43441_2026_982_MOESM2_ESM.docx]

Figure S1 Traceplot of bayesian model estimation
